# Therapeutic Efficacy and Factors Associated With Acceptance of a Newly Developed Compression Garment in Patients With Lower-Extremity Edema

**DOI:** 10.7759/cureus.82967

**Published:** 2025-04-25

**Authors:** Yukako Ishida, Mitsuyo Kikutani, Yoko Tagawa, Fuminori Kimura, Akira Kido

**Affiliations:** 1 Department of Rehabilitation Medicine, Nara Medical University, Kashihara, JPN; 2 Lymphedema Clinic, Nara Medical University Hospital, Kashihara, JPN; 3 Department of Obstetrics and Gynecology, Nara Medical University, Kashihara, JPN

**Keywords:** acceptance, compression garment, compression therapy, lymphedema, venous edema

## Abstract

Background

The principle of edema treatment is compression therapy. We recently reported the development of a new compression garment designed to maintain a high pressure that is affected only to a small extent by changes in posture. In this study, we measured the pressure on this new product in patients with edema and conducted a questionnaire survey to assess its acceptability.

Methods

This is a retrospective observational study that uses existing data from healthy participants as a comparison. We investigated the effect of posture and movement on pressure when wearing the new product in six patients, compared the results with previous data from healthy participants, and statistically analyzed them. In addition, we administered a questionnaire survey on pressure, slipperiness, ease of wearing, and texture.

Results

Similar to the results in healthy participants, the effects of posture and movement were not observed in patients with edema. In contrast, the pressure was significantly lower than that in healthy individuals in the standing, dorsiflexion of the ankle joint, flexion of the toes, and tiptoe positions. The questionnaire suggested that there were issues with the ease of putting on and taking off and that there was a possibility of a discrepancy between the pressure actually measured and the pressure experienced by the participant.

Conclusion

In patients with edema, there are differences in the effects of posture and movement on the measured pressure compared to healthy individuals, which is thought to be due to excess extracellular fluid. However, the required pressure value was achieved, and this new garment could be a possible therapeutic approach with a higher therapeutic effect. The questionnaire results showed some cases in which there was a discrepancy between the patient's evaluation and the actual measurement values, suggesting the need for a quantitative and objective evaluation.

## Introduction

Edema is a condition in which excess fluid accumulates in interstitial tissues. It can be classified into two types: systemic and local [1-3]. The former is caused by heart failure or renal failure, and disorders of the local veins or lymph vessels cause the latter. Local edema includes lymphoedema, caused by dysfunction of the lymphatic system [1-3], and venous edema, caused by chronic venous insufficiency [3,4]. Lymphedema often occurs after surgery in gynecological neoplasms, and over 90% of patients are female [1,2]. This edema is progressive and intractable and can be accompanied by cellulitis and ulcers, leading to a decline in activities of daily living and quality of life. The primary treatment for edema is conservative therapy. The main reason for this was compression therapy. It involves the use of elastic bandages, stockings, and other elastic garments. It is recommended that a compression pressure of 15 mmHg or more be used for venous edema of the ankle joint [3,5,6] and a pressure of 20-60 mmHg for lymph edema [2]. The Japanese Society of Lymphology recommends a complex decongestive therapy consisting of compression therapy using elastic stockings, exercise therapy, lymphatic drainage, and skin care as conservative treatment for lymphedema [7]. 

In September 2016, our hospital established a lymphedema outpatient clinic, and treatment was mainly provided by two nurses qualified as specialized lymphatic drainage therapists. In 2023, we began the treatment with “calf supporters” (Takagi, Nara, Japan), a simple compression garment for the lower legs [8]. This product is a fully-sewn, seamless, Velcro®-type supporter. Although this product showed a certain degree of clinical efficacy, patients commented that (i) choosing the right size was difficult and (ii) gaps between the pullers made the pressure application difficult, causing the garment to slip. As part of a joint industry-academia research project between our university and the manufacturer, we developed a new garment shape that reflects the opinions of patients visiting lymphedema outpatient clinics. We attempted to improve the compression garments already in use for treatment. The preliminary results of our study on the pressure effects of this new garment in seven healthy participants have already been reported [8]. In this report, we clarified that our new product maintains a higher pressure than current products in healthy individuals and is unaffected by posture or movement. Next, in this study, we aimed to investigate the effects of this new product on patients with a pathological interstitial environment due to edema.

Factors affecting the pressure exerted by garments include those on the body and garments. The former includes posture and movement, whereas the latter includes the shape of the pressure garment, which is the subject of this study, and the stretching properties of the materials. When comparing products with different degrees of elasticity, posture affects all cases; however, products with lower elasticity are preferable [9-11].

However, it is not difficult to imagine that the mechanical properties of interstitial tissue in which excessive fluid retention occurs are very different from those of healthy interstitial tissue. In addition, because compression garments are designed to be worn for long periods in everyday life, patient acceptance is also an essential factor from the perspective of treatment compliance [12-14]. In other words, the development of new compression garments that are more acceptable to patients and that can maintain higher pressures will make it possible to improve current treatment methods. Therefore, in this study, we focused on the factors related to the compression effect and treatment compliance of a newly developed compression garment in actual clinical settings.

## Materials and methods

Participants

Patient selection criteria were as follows: patients who attended the lymphedema outpatient clinic once a month or once every two months were able to perform standing exercises while wearing compression garments and were able to complete a questionnaire regarding the use of compression garments. Exclusion criteria were as follows: patients who were unable to perform standing exercises due to cognitive impairment or other reasons, or who were unable to complete the questionnaire. Six female patients with lower-extremity edema were enrolled in the study. The ages of the participants ranged from 63 to 83 years (mean age 73.3 ± 8.0). The purpose and content of the study were explained to each participant, and their cooperation was requested after they understood the details. We explained that participation in the study was based on their own free will and obtained their written consent. In addition, we compared the pressure data of seven healthy individuals from our database as controls. The use of the database for patients and healthy people and the publication of the research results were approved by the Nara Medical University Ethics Committee (research approval number 3976). The study was conducted between April 1, 2023 and February 28, 2025.

Compression garment

The product, “calf supporter” (Takagi Co., Nara, Japan), is a Velcro®-type supporter that can be adjusted for pressure by wrapping the puller around the lower leg. The outer fabric is composed of 87% nylon and 13% polyurethane, whereas the inner fabric is composed of 95% nylon and 5% polyurethane. The surface that touches the skin is covered with satin net for a good feel against the skin, and the feature is that the seams do not touch the skin because it is completely sewn without seams. We previously reported that the number and shape of compression garment pullers are important factors affecting compression [8]. The shape of this newly developed product was based on feedback from patients at our hospital's lymphedema outpatient clinic from 2021. It has been shown to have an ideal compression effect in healthy people, which is significantly better than conventional products and is not affected by posture or movement [8]. In this study, we evaluated this new product in patients with lower-extremity edema. Two registered nurses who were certified lymphedema drainage therapists instructed the patients on application of the compression garment at the appropriate pressure, using the measured pressure and physical findings as indicators.

Measurement items

The measurement items included height, weight, body mass index (BMI), grip strength, lower-leg circumference, extracellular water-to-total body water ratio (ECW/TBW), and pressure while wearing a supporter. Lower-leg circumference was measured as the maximum circumference of the lower leg. InBody S10 (InBody Japan, Tokyo, Japan) was used to measure the ECW/TBW. PicoPress® (Microlab Elettronica, Ponte San Nicolò, Italy) was used to measure the interface pressure between the body and the garment. The sensor was placed in the transition area between the Achilles tendon and gastrocnemius muscle. Measurements were taken in the following positions: supine, standing, squatting (knee joint flexion 30°), standing with the ankle joint dorsiflexed, standing with the toes flexed, and standing on the toes. For the supine and standing positions, the posture was maintained until the pressure stabilized, and the most stable 10-second value was averaged. For squats, ankle dorsiflexion, toe flexion, and tiptoe, each exercise was performed five times, and the values were averaged.

Questionnaire survey

The patient was prescribed a new supporter, instructed by a nurse on application, had their pressure measured, and after exercise instruction by a physiatrist, was asked to complete a questionnaire at a follow-up visit after wearing it for 3-4 weeks. Questionnaire items were collected using a Likert scale, and four questions were asked about pressure, ease of slipping, ease of wearing, and texture. In addition, we asked if assistance was required in wearing, the number of hours it was worn, whether a base was used, and whether skin problems were encountered (Table 1).

**Table 1:** Questionnaire on the use of calf supporters. Patients with lymphoedema visit the outpatient clinic every three to four weeks. This questionnaire was conducted at the next consultation after the first prescription of the new supporter.

Age range □10s □20s □30s □40s □50s □60s or older
1. How did you feel about the pressure from the supporters?
□Very good □Fairly good □Can't say either □Not very good □Not good at all
2. Were the supporters easy to slip
□Very difficult to slip □Fairly difficult to slip. □Can't say either □Fairly easy to slip □Very easy to slip
3. Was it easy to put on the supporter?
□Very easy to put on □Somewhat easy to put on □Neither easy nor difficult □Somewhat difficult to put on □Very difficult to put on
4. How was the texture of the supporter?
□Very good □Somewhat good □Neither good nor bad □Not very good □Not good at all
5. Who put on the supporter?
□Myself □Family member. □Medical professional □Other ( )
6. How long did you wear the supporter each day?
( ) hours
7. Did you use a base for the supporter?
□Yes □No
8. Did you experience any skin problems while using the supporter or after using it?
□No □Yes ( )
9. Did you experience any changes in your condition (swelling, fatigue, etc.) before or after using the supporter?
□No □Yes ( )

Statistical analysis

The Mann-Whitney U test was used to compare the measurement positions of the new products and the pressure in the same posture between healthy individuals and patients. Statistical significance was set at P < 0.05. Data were analyzed using JMP Pro version 17.2.0 (SAS Institute Inc., Cary, NC, USA).

## Results

Clinical characteristics of the patients

**Figure 1:**
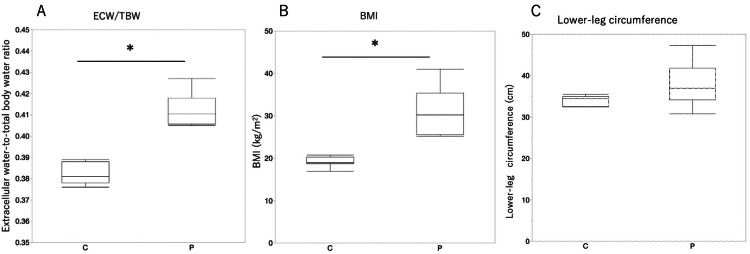
Comparison of ECW/TBW (A), BMI (B), and lower-leg circumference (C) between the healthy individuals (C) and patients (P). The Mann–Whitney U test was used to compare the measurement values. *p<0.05 ECW/TBW: Extracellular water-to-total body water ratio

The clinical characteristics of the patients are summarized in Table 2. The average height was 146.0±7.3cm, the average weight was 66.2 ± 14.5 kg, and the average BMI was 31.0 ± 5.4 kg/m^2^, corresponding to a degree 2 obesity classification by the Japanese Society for the Study of Obesity. The average lower-leg circumference on the side where the device was attached was 37.9±5.1 cm. The average ECW/TBW was 0.412 ± 0.007, which was higher than the reference value. Two patients were diagnosed with venous edema, and the remaining four were diagnosed with lymphedema and venous edema. The BMI, lower-leg circumference, and body composition were compared with the data of seven healthy individuals obtained from our database. The clinical and pathological characteristics of the healthy subjects reported previously were as follows: mean age (29.7 ± 7.3 years), mean height (160.2 ± 3.7 cm), mean weight (49.4 ± 3.8 kg), and mean BMI (19.3 ± 1.2 kg/m^2^). The average lower-leg circumference (right lower leg) on the side where the device was attached was 34.0 ±1.2 cm. The average ECW/TBW was 0.382 ± 0.004, which is within the reference range. The values for patients were significantly higher when ECW/TBW and BMI were compared between patients and healthy individuals. However, there was no difference in the lower-leg circumference (Figure 1).

**Table 2:** Patient characteristics. * The diagnosis was made comprehensively based on physical examination findings, radiographic findings, and lymphatic echo findings. ** When edema was observed on both sides, the side with more severe symptoms was the subject of the study.

Patient No.	Diagnosis*	Age	Height	Weight	Side**	BMI	Circumference of the lower leg(rt/lt)	Body composition
1	Venous edema	82	143.4	59.5	Left	29.0	32.5/30.8	0.408
2	Venous edema	74	147.0	68.0	Left	31.5	37.9/38.0	0.415
3	Lymphedema + venous edema	63	154.0	59.8	Left	25.2	41.2/40.0	0.427
4	Lymphedema + venous edema	63	153.9	97.2	Right	41.0	47.3/48.2	0.406
5	Lymphedema + venous edema	83	132.4	58.8	Right	33.5	35.9/35.5	0.413
6	Lymphedema + venous edema	75	145.0	54.0	Right	25.7	35.2/34.4	0.405

Interface pressure measurement

**Figure 2:**
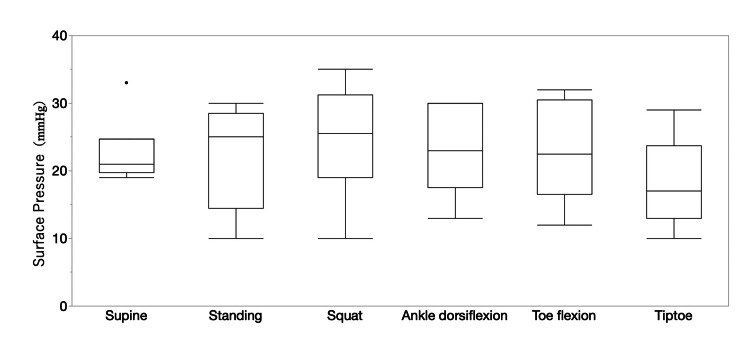
Interface pressure in the supine position, standing position, and during movement. The Mann–Whitney U test was used to compare the measurement values.

**Figure 3:**
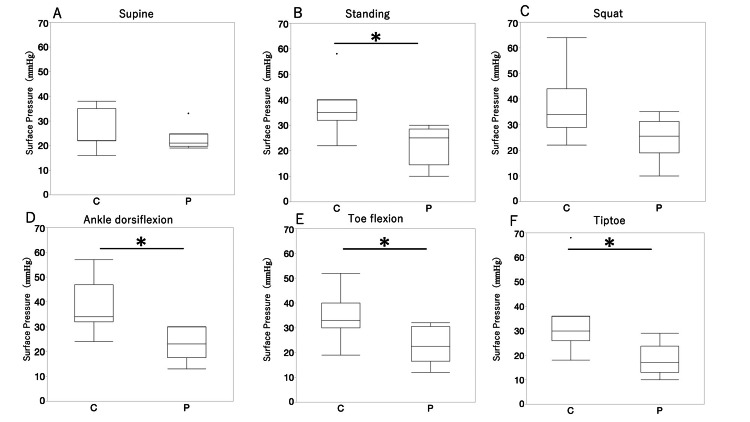
Comparison of the interface pressure between the healthy controls (C) and the patients (P). The Mann–Whitney U test was used to compare the measurement values in supine (A), standing (B), squatting (C), standing with ankle dorsiflexed (D), standing with toes flexed (E), and standing on toes (F). *p<0.05

The results of patient pressure measurements are shown in Figure 2. The pressure in the supine position was 22.67 ± 4.71 mmHg, the pressure in the standing position was 22.33 ± 7.25 mmHg, the pressure during the squat exercise was 24.67 ± 7.99 mmHg, the pressure in the standing position with dorsiflexion of the ankle joint was 23.0 ± 6.08 mmHg, the pressure in the standing position during toe flexion was 22.83 ± 6.84 mmHg, and the pressure when standing on tiptoes was 18.17 ± 6.15 mmHg. The pressure when standing on the tiptoes was the lowest, and the pressure when squatting was the highest; however, there was no significant difference in the pressure in the supine position or in the pressure in other postures. Figure 3 shows the comparison with healthy individuals. The pressure in the supine position was 24.6 ± 7.31 mmHg, the pressure in the standing was 32.2 ± 5.64 mmHg, the pressure during squatting was 37.43 ± 12.70 mmHg, the pressure during standing with the ankle in dorsiflexion was 38.71 ± 10.50 mmHg, the pressure during toe flexion was 35.0 ± 9.56 mmHg, and the pressure during tiptoe standing was 34.29 ± 14.86 mmHg. When comparing pressure in the same posture between healthy people and patients, the pressure in healthy people was significantly higher in the standing, ankle dorsiflexion, toe flexion, and tiptoe positions.

Questionnaire survey

Table 3 presents the results of the questionnaire. The first four items in the questionnaire were collected using the Likert scale, and the items were about pressure, ease of slipping, ease of wearing, and texture. We assigned scores from 1 to 5 to each item, ranging from most desirable to least desirable. Five of six participants gave scores of 1 or 2 for the two items of pressure and texture, which we considered relatively good. For the “does not slip” item, three out of the six participants scored 1 or 2, while one gave a score of 5, suggesting a problem compared to the two items mentioned earlier. For the question on ease of wearing, only two people gave a score of 2, two gave a score of 3, and two gave a score of 4 or 5, suggesting a significant issue with this garment. Although many of the responses from Patient 2 were not ideal, the measured interfacial pressure showed promising results, indicating the importance of providing quantitative and objective evaluations to patients to maintain appropriate treatment effects.

**Table 3:** Questionnaire results. The first four items in the questionnaire were collected using the Likert scale. We assigned scores from 1 to 5 to each item, ranging from most desirable to least desirable.

Patient No.	Pressure	Slip	Ease of wearing	Texture	Wearer	Time (hours/day)	Base use	Skin problems
1	2	2	4	2	Myself	12	Yes	No
2	4	5	5	3	Myself	5-7	Yes	No
3	1	3	2	1	Myself	5	Yes	No
4	2	1	3	1	Myself	5-6	Yes	No
5	1	1	2	2	Myself	12	Yes	No
6	2	3	3	2	Myself	2-5	Yes	No

## Discussion

In this study, we measured the pressure of a newly developed compression garment in patients with lower-extremity edema and conducted a questionnaire survey on acceptance. The results of the measurement of the interface pressure were consistent with the results of the measurement of healthy individuals that we have already reported, and there were no significant differences in the results of this measurement due to posture or movement. As noted in our previous study, the shape of this new product is less affected by posture and movement than conventional products. In contrast, when compared with healthy individuals, the results for standing, ankle dorsiflexion, toe flexion, and tip-toe were significantly lower than those for healthy people. However, no significant difference was observed between the supine and squatting positions. Owing to the small sample size, it was impossible to make an overly optimistic assessment of the presence or absence of differences in posture or type of movement. However, it is possible that the mechanical properties of the interstitial tissue, where excessive fluid retention occurs, could cause differences in the measurement data for healthy people. In addition, the measurement results were higher than the 20 mm Hg required for treatment reported in the literature [2,5,6]. Extrapolating from the experience with the old product of the identical material, which has been used in the lymphedema outpatient clinic since 2021, a certain compression effect can be expected.

The importance of therapeutic exercises in compression therapy has been reported previously [1-3]. The aim is not simply to increase pressure through exercise, but to contract the smooth muscle of the walls of the lymphatic transport tubes and establish an internal pumping mechanism [11,15-17]. Furthermore, several groups have also reported periodic pressure changes during exercise when wearing elastic clothing [5,9,10]. Although evidence for this is still empirical, there is high hope for its use in combination with therapeutic squatting exercises [18].

The wear time is an extremely important factor in addition to the effects of posture and movement [19,20]. Compression garments are designed on the premise that they will be worn for long periods in everyday life based on their therapeutic mechanism; therefore, from the perspective of treatment compliance, patient acceptance is also considered an extremely important factor [12-14]. In this study, a questionnaire survey was administered to address these issues. Among the items collected using the Likert scale, the results suggested that there were issues with ease of wearing rather than pressure or texture. Interestingly, while one patient responded with undesirable intentions for multiple items, including pressure, the measured pressure of this patient presented an ideal value, suggesting that it is essential to show patients a measurable evaluation system to appropriately maintain the therapeutic effect.

This study has several limitations. First, the sample size is small. Second, when comparing the data with those of healthy individuals, we used existing data from a significantly different age group. However, we have already reported comparisons between posture and movement in the existing data, which were also helpful in interpreting the current data. We believe that lower-extremity edema is a heterogeneous condition and that compression therapy is also influenced by empirical factors more than drug therapy or surgical therapy. In treatments involving garments, feedback from everyday life experiences is likely to be as important as a theoretical basis based on measurements. Our research series is an industry-academia collaborative research project aimed at improving conventional products that have already been used clinically. Therefore, one of its strengths is that it is not a fragile prototype but has sufficient durability to withstand practical use. We plan to conduct clinical research with a larger sample size in the near future using the data from this study as basic information.

## Conclusions

We measured the pressure of a newly developed compression garment in patients with lower-extremity edema and conducted a questionnaire survey on their acceptance of the garment. The interface pressure was maintained to a large extent and a therapeutic effect was expected. In addition, there were no significant differences in posture or during movements; this was the same result as the measurements in healthy people that we previously reported. During some movements, it was difficult to increase the pressure using this product. There is a possibility that this is due to the involvement of an excess of extracellular fluid. However, in the supine position and squatting, the pressure was the same as that in healthy people, and a therapeutic effect could be expected under these conditions.
